# Electromagnetic Vibration Energy Harvester with Tunable Resonance Frequency Based on Stress Modulation of Flexible Springs

**DOI:** 10.3390/mi12091130

**Published:** 2021-09-20

**Authors:** Yunjia Li, Chenyuan Zhou, Qi Cao, Xinyi Wang, Dayong Qiao, Kai Tao

**Affiliations:** 1 School of Electrical Engineering, Xi’an Jiaotong University, Xi’an 710049, China; zhouchenyuan@stu.xjtu.edu.cn (C.Z.); caoqisky@126.com (Q.C.); xywang@stu.xjtu.edu.cn (X.W.); 2 Micro and Nano Electromechanical Systems Laboratory, Northwestern Polytechnical University, Xi’an 710072, China; dyqiao@nwpu.edu.cn

**Keywords:** polymer beam, vibration energy harvesting, tunable resonant frequency

## Abstract

This paper presents a compact electromagnetic vibrational energy harvester (EVEH) with tunable resonance frequency. The resonance frequency of the EVEH is tuned by adjusting the axial stress in the flexible polymeric springs, which is realized by physically pulling and pushing the springs. The stress tuning functionality is realized with a compact structure with small volume. The total frequency tuning range of the proposed EVEH is 56 Hz (74–130 Hz), which is 64% of the natural resonance frequency of the EVEH (88 Hz). It is found that the tensile stress increases the resonance frequency of the EVEH, while the compressive stress firstly reduces the resonance frequency and then increases the resonance frequency due to buckling.

## 1. Introduction

Electromagnetic vibration energy harvesters (EVEHs) are devices capable of converting vibration kinetic energy to electrical power [1,2]. The transduction process usually involves relative movement between a permanent magnet and a solenoid coil induced by external vibration. Most of the reported EVEH devices are resonant structures that output maximum power only when its mechanical resonance frequency matches the external vibration frequency. Under off-resonance conditions, the output power of the EVEH decreases significantly. However, the frequency matching is often difficult because the frequency of the environmental vibration sources is often random and unpredictable [3]. Even if the source vibration frequency is constant, it is still difficult to realize the frequency matching due to uncertainties and inaccuracies of the fabrication processes and geometrical parameters of the devices [4].

Two major approaches to improve the frequency matching between the VEH and the vibration sources are the frequency tuning and band broadening techniques. The frequency tuning techniques can be realized by both mechanical tuning and electrical tuning approaches. Mechanical tuning approaches can be implemented by different structures and technologies, but the basic principle is mainly based on tuning the resonance frequency of the VEH by changing certain physical parameters of the device such as the mass or the stiffness [5,6]. Electrical tuning approaches is mainly based on load tuning or frequency tracking technologies. However, Roundy et al. [7] showed that for the active frequency tuning techniques, the energy consumed by the tuning actuators is sometimes more than the energy generated by the VEH, which limits the application of these approaches. The frequency band broadening techniques can be realized by four main different configurations: multi-mode devices, multi-frequency devices, nonlinear devices and bistable devices. The multi-mode device uses different resonance modes of the VEH to harvest energy in different frequency bands [8,9,10,11,12]. Nevertheless, the operation of this type of device is still limited to several rather narrow frequency bands. To extend the number of these bands, the VEH can be implemented using array of structures with different size to realize multi-frequency operation [13,14,15]. These bandwidth tuning techniques above extend the frequency band of the VEHs to several fixed bands. However, for excitation vibrations with arbitrary frequencies, it is still possible that the VEH’s operation band mismatches the excitation frequency. In addition to the multi-mode or multi-frequency methods, the frequency band of the VEH can also be broadened by utilizing nonlinear springs. It has been shown that the cubic stiffness of the springs effectively broadens the bandwidth of the VEH [16,17]. However, one of the deficiencies of the nonlinear VEH is the hysteresis in the frequency response, i.e., different sweep-up and sweep-down characteristics in the frequency domain. In comparison, the bistable broadband energy harvester has a better-defined frequency response to vibrations, but the excitation vibration must be beyond certain threshold to reliably operate this type of device.

From the literature above, it can be concluded that the main goal for the frequency tuning technologies is large tuning range or wide bandwidth. However, the working frequency range of many reported works are still limited to around 10 Hz. In addition, many of the proposed devices are still proof-of-concept prototypes which are rather bulky in size. In this paper, we propose a compact EVEH device with large tunable resonance frequency, based on soft polymer springs and stacked polymer coils. The frequency tuning function of the EVEH is realized by a compact tuning structure that changes the stress within the polymeric springs. The stacking of repeating thin polymer coils enables high output voltage of the EVEH device and the resonance frequency of the EVEH is tuned by modulating the axial stress in the polymer springs. The remainder of the paper is organized as follows: Section 2 reports the design of the EVEH device, Section 3 describes the experimental procedures of the work, Section 4 presents the results and discussions and Section 5 is the conclusion of the work.

## 2. System Design

### 2.1. Concept of the Device

**Figure 1:**
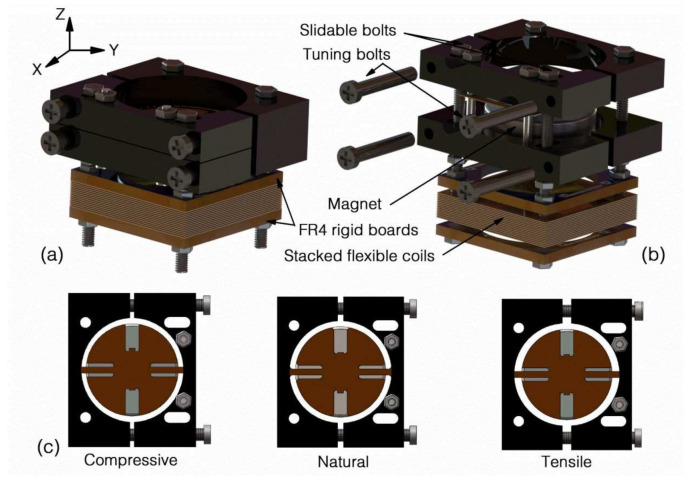
Schematically illustration of (**a**) the assembled EVEH device; (**b**) the EVEH in an exploded view; and (**c**) tuning process of the EVEH.

The proposed EVEH is schematically illustrated in Figure 1a,b. A disc magnet is suspended by two straight polyimide springs over a stack of flexible coil layers. The details of the flexible coils are detailed in another work [17]. When the EVEH is subjected to external vibration, the magnet oscillates and generates a changing magnetic field around the coil. Consequently, electrical power is generated in the coil via induction. To minimize the footprint of the device, the disc magnet is glued onto a 1 mm-thick reinforcement structure which provides space for the springs to oscillate. The polyimide springs are laser-cut straight beams with a length of 5.5 mm, a width of 1 mm and a thickness of 0.16 mm. Each of the springs is connected to an anchor frame, which is sandwiched between two anodized aluminum fixtures (four fixtures in total for the two anchors). Among the four aluminum fixtures, two are fixed to the stacked coils and two are free to move along the X direction in Figure 1. The four bolts on the side of the two movable aluminum fixtures can be fastened or loosened to adjust the distance between the movable fixtures and the fixed fixtures. By adjusting the distance, compressive or tensile stress is introduced into the polyimide springs, resulting in the shift of the EVEH’s resonance frequency, as shown in Figure 1c. Once the tuning is complete, the position of the movable fixture is fixed by two slidable bolts in the trenches. The other two fixed bolts visible on top of the movable fixture are used to create a firm and strong clamping force for the anchor area of the polyimide springs. During the assembly of the EVEH device, acrylic glue is used between the polyimide anchor and the fixtures to further improve the clamping and facilitate the assembly process. The coils used in the study are a stack of 15 layers of double-sided copper planar coils electroplated on 180 μm-thick polyimide substrates. The flexible coil layers are clamped between two rigid FR4 frames to be electrically interconnected, forming a serially connected high density coils.

### 2.2. Analytical Modeling

For a uniform spring under small deflections, if we assume the axial load is constant, the equation of motion can be obtained by summing the internal shear, moment and inertial forces acting on an element d*x* in the spring length. Therefore, the equation of motion for the spring under constant axial load *F* is given by [18]:

(1) $$\frac{\partial^{4}V\left(x,t\right)}{\partial x^{4}}+\frac{F}{EI}\frac{\partial^{2}V\left(x,t\right)}{\partial x^{2}}+\frac{\rho }{EI}\frac{\partial^{2}V\left(x,t\right)}{\partial t^{2}}=0,$$

where *V*(*x*, *t*) is the deflection of the spring at the position *x* and time *t*. *F* is the axial load. *E* and *I* are the Young’s modulus and the area moment of inertia of the spring’s cross section, respectively and *ρ* is the mass per length. Assuming that the spring performs harmonic oscillations with an angular frequency *ω*, the expression for its transversal displacement can be written as:

(2) $$V\left(x,t\right)=v\left(x\right)\sin\left(\omega t\right),$$

Substituting Equation (2) into Equation (1) yields:

(3) $$\frac{d^{4}v\left(x\right)}{dx^{4}}+k^{2}\frac{d^{2}v\left(x\right)}{dx^{2}}-\beta^{4}v\left(x\right)=0,$$

where *k*^2^ = *F*/*EI* and *β*^4^ = *ρω*^2^/*EI*, *v*(*x*) describes the oscillation amplitude of the spring at position *x*. Transforming this equation into a nondimensional form by letting *x* = *x*/*l*, *v* = *v*/*l*, *k* = *kl* and *β* = *βl*. The nondimensional equation of motion is then given by:

(4) $$\frac{d^{4}\bar{v}\left(\bar{x}\right)}{d{\bar{x}}^{4}}+{\bar{k}}^{2}\frac{d^{2}\bar{v}\left(\bar{x}\right)}{d{\bar{x}}^{2}}-{\bar{\beta }}^{4}\bar{v}\left(\bar{x}\right)=0$$

The solution to this differential equation is given by:

(5) $$\bar{v}\left(\bar{x}\right)=A\cos h\left(\alpha_{1}\bar{x}\right)+B\sin h\left(\alpha_{1}\bar{x}\right)+C\cos\left(\alpha_{2}\bar{x}\right)+D\sin\left(\alpha_{2}\bar{x}\right),$$

where *A*, *B*, *C* and *D* are constants that can be obtained from certain boundary conditions and the constants *α*_1_ and *α*_2_ are given by:

(6) $$\begin{array}{c} \alpha_{1}=\sqrt{-\frac{{\bar{k}}^{2}}{2}+\sqrt{\frac{{\bar{k}}^{4}}{4}+{\bar{\beta }}^{4}}}\\ \alpha_{2\;}=\sqrt{\frac{{\bar{k}}^{2}}{2}+\sqrt{\frac{{\bar{k}}^{4}}{4}+{\bar{\beta }}^{4}}} \end{array}$$

**Figure 2:**
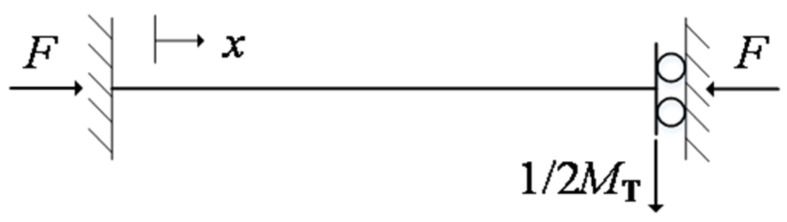
Schematic diagram of the clamped-guided spring with tip mass.

The spring-magnet structure in this work can be modeled as a clamped-guided beam with tip mass, as shown in Figure 2. The equivalent tip mass is half of the mass of the magnet and reinforcement structure. The boundary conditions of the spring are given as follows:

(7) $$\begin{array}{cc} \begin{array}{c} \bar{v}\left(0\right)=0\\ {\bar{v}}^{\prime }\left(0\right)=0 \end{array} & \mathrm{Clamped}\\ \begin{array}{c} {\bar{v}}^{\prime }\left(1\right)=0\\ {\bar{v}}^{\prime \prime \prime }\left(1\right)+{\bar{k}}^{2}{\bar{v}}^{\prime }\left(1\right)+\frac{1}{2}\eta_{T}{\bar{\beta }}^{4}\bar{v}\left(1\right)=0 \end{array} & \mathrm{Guided}\;\mathrm{with}\;\mathrm{tip}\;\mathrm{mass} \end{array},$$

where *η*_T_ is the ratio between the tip mass *M*_T_ to the spring mass *M* = *ρl*.

(8) $$-\eta_{T}{\bar{\beta }}^{4}\alpha_{1}+\frac{1}{2}\eta_{T}{\bar{\beta }}^{4}\left(\frac{\alpha_{2}^{2}-\alpha_{1}^{2}}{\alpha_{2}}\right)\sin h\left(\alpha_{1}l\right)\sin(\alpha_{2}l)\;+\eta_{T}{\bar{\beta }}^{4}\alpha_{1}\cos h\left(\alpha_{1}l\right)\cos\left(\alpha_{2}l\right)+\left(\alpha_{1}^{2}\alpha_{2}^{2}+\alpha_{1}^{4}\right)\sin h\left(\alpha_{1}l\right)\cos\left(\alpha_{2}l\right)\;+\left(\alpha_{1}^{3}\alpha_{2}+\alpha_{1}\alpha_{2}^{3}\right)\cos h\left(\alpha_{1}l\right)\sin\left(\alpha_{2}l\right)=0$$

**Figure 3:**
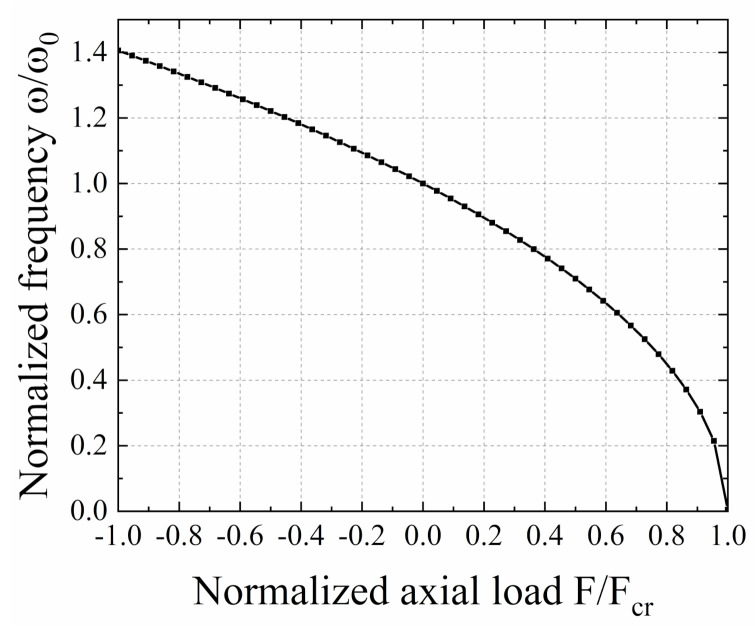
The analytical normalized frequency *ω*/*ω*_0_ as function of normalized axial load *F*/*F*_cr_.

The design and structural parameters of the proposed EVEH is given in Table 1. By using these values, the analytical solution of Equation (8) can be obtained. The normalized frequency *ω*/*ω*_0_ as a function of normalized axial load *F*/*F*_cr_ is shown in Figure 3. When the normalized axial load is 0, the normalized frequency of the device is defined as 1. Under compressive load (*F*/*F*_cr_ is positive), *ω*/*ω*_0_ decreases as the compressive load increases. When the critical buckling point (*F*/*F*_cr_ = 1) is reached, *ω*/*ω*_0_ drops to zero theoretically. Under tensile load (*F*/*F*_cr_ is negative), ω/ω_0_ increases as the tensile load increases. The maximum value of *ω*/*ω*_0_ is 1.406, at a *F*/*F*_cr_ value of −1. This indicates that when the critical buckling load is applied, the resonance frequency of the EVEH device is 40.6% of the natural resonance frequency of the device. In another word, by adjusting the axial load in the spring, a normalized frequency tuning range of 40.6% with respect to the natural resonance frequency of the EVEH device can be achieved.

### 2.3. Finite Element Method (FEM) Modeling

**Figure 4:**
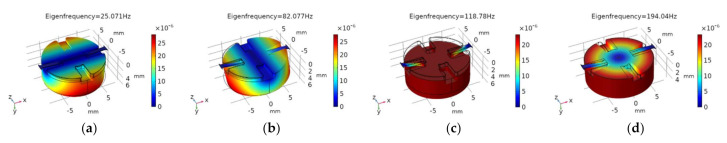
FEM Modal analysis of the EVEH. (**a**) twisting around the *x*-axis (25.07 Hz); (**b**) twisting around the *z*-axis (82.08 Hz); (**c**) out-of-plane piston (118.78 Hz); (**d**) in-plane twisting around the *y*-axis (194.04 Hz).

A FEM modal analysis of the EVEH is performed with Comsol Multiphysics. In the model, the anchoring frame structure is simplified by applying fixed constrains to the end of the springs. This simplification is valid as the springs are fixed at the end when being clamped between two rigid frames. Different axial loads are asserted by specifying different axial displacements of the end of the springs. The material parameters of the polyimide used in the simulation are Young’s Modulus of 3.2 GPa, Poisson ratio of 0.34. The four primary vibrational modes of the EVEH are shown in Figure 4: out-of-plane twisting mode around the *x*-axis at 25.07 Hz, out-of-plane torsional mode around the *z*-axis at 82.08 Hz, the out-of-plane piston motion mode at 118.78 Hz and in-plane twisting mode around the *y*-axis at 194.04 Hz. In spite of being the 3rd resonance mode, the piston motion mode will be the dominant resonance mode during operation as the entire EVEH device is subjected to a uniform acceleration vertically (Y-direction in Figure 4). Slight torsional movement may be combined the piston motion movement of the proof mass due to the slight geometrical asymmetry induced by the assembly process of the device, e.g., the off-centered position of the magnet induced by the gluing process.

**Figure 5:**
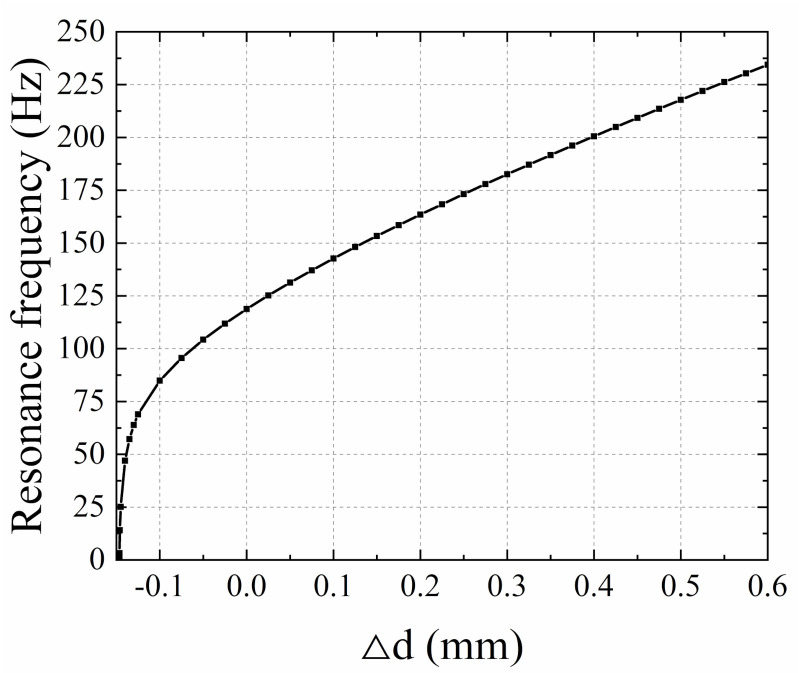
The FEM resonance frequency as function of the relative fixture distance ∆d.

For the piston mode of the EVEH device, the effect of the axial stress on the resonance frequency is studied. The resonance frequency as a function of the relative fixture distance ∆d is shown in Figure 5. The resonance frequency of the device with no stress in the spring is 118.78 Hz. When tensile force (∆d > 0) is applied, the resonance frequency increases with the increase of ∆d. In the simulation, a maximum resonance frequency of 234.46 Hz is obtained with ∆d of 0.6 mm. When compressive stress (∆d < 0) is applied, the resonance frequency decreases rapidly with the increase of stress. It is difficult to predict the influence of spring buckling on the resonance behavior of the EVEH. The simulated resonance frequency of the EVEH drops to 0 at the critical buckling point of ∆d = −0.146 mm. However, it has been shown that buckling stiffens the spring because it changes the resonant movement of spring from dynamic bending to dynamic stretching [19]. Therefore, as ∆d further decreases beyond the buckling mode, the spring-stiffening effect originated from the tensile stress induced by buckling will begin to dominate and the resonance frequency will rise accordingly.

## 3. Experimental

**Figure 6:**
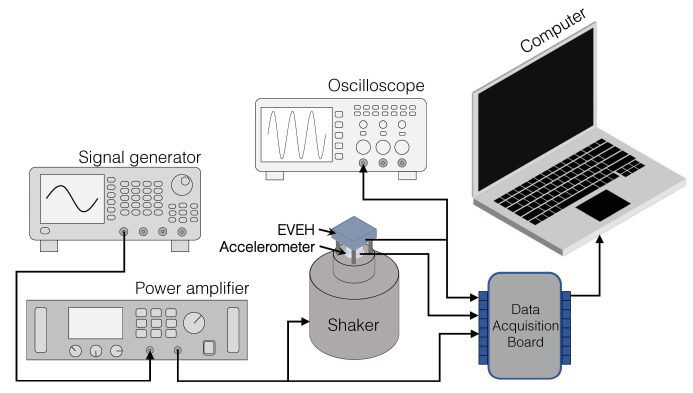
Schematic diagram of the measurement setup.

Figure 6 shows the measurement setup for characterizing the EVEH. The setup is capable of generate different vibration conditions while simultaneously measuring the output voltage of the EVEH device. The assembled EVEH is tuned to different resonance frequency and then systematically characterized when being excited by a piezo shaker. The sinusoidal actuation signal of the shaker is generated by a signal generator and amplified by a power amplifier. The proposed EVEH has FR4 rigid PCB board at bottom which facilitates the mounting process. The acceleration applied to the EVEH is measured by an accelerometer installed between the EVEH and the shaker. The signals of the EVEH and the accelerometer are measured simultaneously using a NI USB-6211 data acquisition board. A 4th-order Butterworth low-pass filter is applied to the measured output of the EVEH to filter the high frequency noise. The filter is implemented by using the LabVIEW software and has a cut-off frequency of 1000 Hz which is far beyond the resonance frequency of the EVEH.

## 4. Results and Discussions

This section presents the characterization results of the fabricated tunable EVEH device. As the main goal of the presented work is to demonstrate the frequency tuning capability of the proposed EVEH device, only open-loop performance of the EVEH device in the time and frequency domain is presented, while the impedance matching and closed-loop performance of the EVEH device is not reported in this work.

### 4.1. Manufactured EVEH Device

**Figure 7:**
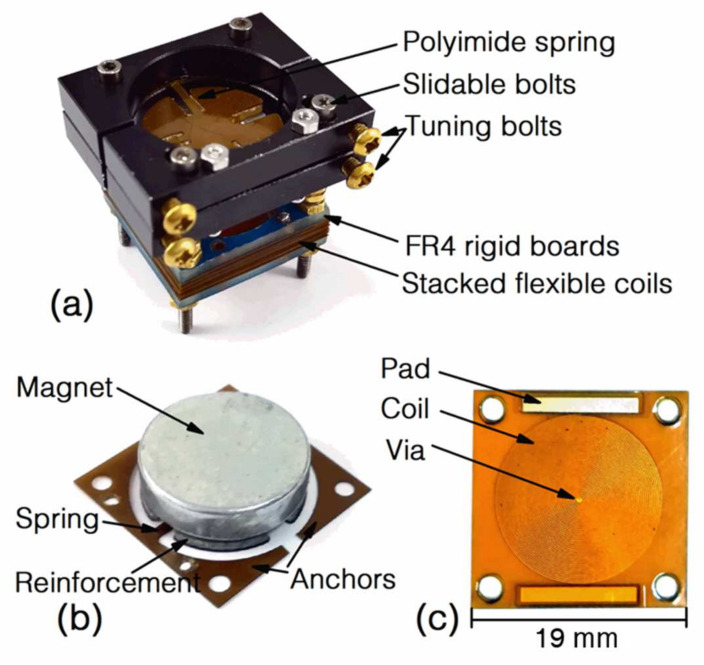
Photos of (**a**) the assembled EVEH device; (**b**) the magnet on the reinforcement structure fixed to the spring layer; and (**c**) a layer of the planar coils.

The photo of the assembled EVEH device is shown in Figure 7a. At the bottom of the device is the stacked flexible coils sandwiched between two rigid FR4 frames. Fifteen layers of 180 μm-thick flexible coils are included in the device shown in the photo. The disc magnet is mounted above the coil stack and suspended by two straight polyimide springs. The anchor of one spring is clamped between two fixed aluminum fixtures, while the anchor of the other spring is clamped between two movable fixtures. The surfaces of the fixtures are black anodized for a better surface protection. The fixtures in the photo are in a slightly compressed state and, thus, the springs are buckled downwards. The magnet in this work is a circular N50 NdFeB magnet with a diameter of 15 mm and a thickness of 4 mm. Figure 7b shows the magnet, the springs and anchors of the EVEH. The magnet is glued onto a reinforcement structure attached to the top surface of the polyimide layer, in order to create space for the springs to oscillate below the magnet and minimize the footprint of the device. Two independent anchors are connected to the end of the springs. When the springs are in a natural state (neither stretched nor compressed), the gap between the magnet and the coil is 4.8 mm. This distance will decrease when the spring is buckled downwards by the compressive stress introduced during the tuning process. Figure 7c shows the photo of a layer of the flexible coils. The coil layer consists of spiral copper wires electroplated on the two sides of a polyimide layer. The patterns of the coils are identical on the two sides but 180 flipped degrees. The winding directions of the coils on the two sides are opposite and are interconnected by the via in the center of the layer. The electrodes at the edge are used to connect coils on different layers. By clamping many coil layers together with 180-degree rotation, a high-density coil consisting of many sub-coils connected in series is formed.

### 4.2. Time Domain Response

**Figure 8:**
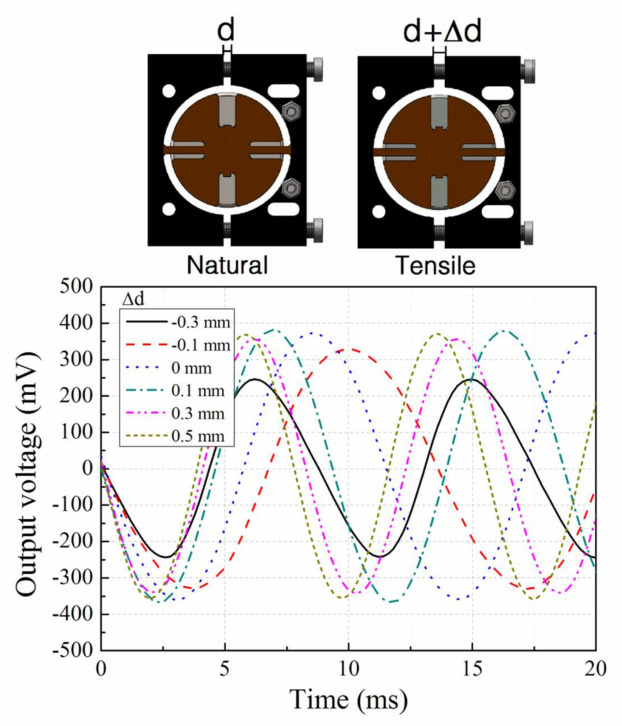
Output voltage of the EVEH versus time, measured at different relative fixture distances ∆d.

The lengths of the springs are tuned by adjusting the distance between the two pairs of fixtures, which is realized by tightening or loosening the side tuning bolts. At each distance, the open-circuit output voltage of the EVEH is measured. The applied excitation signal is a sinusoidal vibration with a peak-to-peak value of 2 g. Figure 8 shows the output voltage of the EVEH as a function of time, measured at the resonance frequency of five different relative distances between fixtures (∆d, with respect to distance between the fixture when the spring is in the natural and relaxed state). These 5 distances correspond to the natural state of the spring (∆d = 0 mm), the extreme compressive state of the spring (∆d = −0.3 mm), the extreme tensile state of the spring (∆d = 0.5 mm) and two intermediate stress levels of the springs (∆d = −0.1 mm and ∆d = 0.3 mm), respectively. The measured output curves have mostly well-defined sinusoidal characteristics with no distortion or nonlinearity, except the curve at ∆d = 0.3 mm. The curve at ∆d = −0.3 mm exhibits slight non-symmetric characteristics, as a result of the buckling-induced nonlinearity from elevated compression stress level. The period of the EVEH’s output voltage corresponds with the excitation vibration frequency. The peak-to-peak output voltage of the EVEH under natural state (∆d = 0 mm) is 749.8 mV. It is lower than the results of 1.57 V we have reported in another work due to the large coil-magnet distance in this work [17]. As the main goal of this EVEH is to tune the resonance frequency, only open-loop performance of the EVEH device is investigated in this work, while the impedance matching and closed-loop performance of the EVEH device is not detailed. The power produced by this device under natural state (∆d = 0 mm) with a matching resistance of 923 Ω is approximately 19.04 μW.

### 4.3. Frequency Tuning of the Device

**Figure 9:**
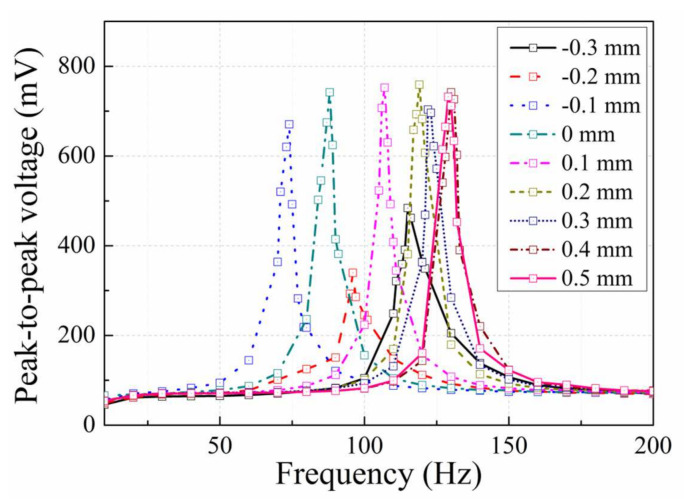
Output peak-to-peak voltage as a function of frequency, measured at different relative fixture distance ∆d.

**Figure 10:**
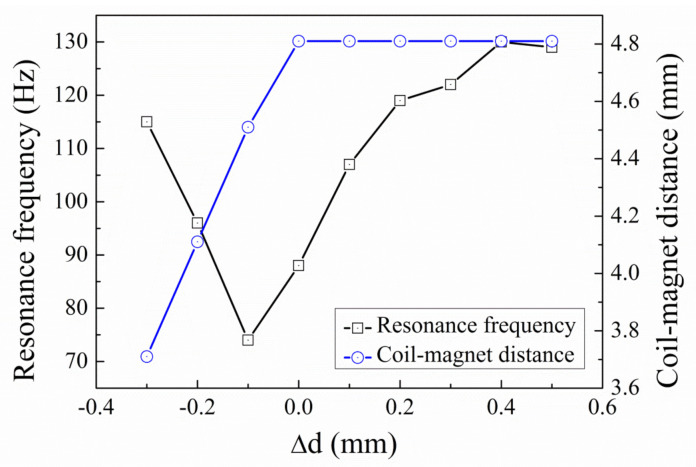
Resonance frequency and coil-magnet distance as a function of relative fixture distance ∆d.

Figure 9 shows the output voltage of the EVEH as a function of the frequency, measured at different fixture distance. All the voltage-frequency curves show typical linear characteristic with symmetric resonance peaks. The total tuning frequency range is 56 Hz (74–130 Hz), which is 64% of the natural resonance frequency of the EVEH (88 Hz). The tuning range of 64% is 3 times higher than the tuning range of 22% realized by an active frequency tuning technique (which consumes energy) of an EVEH with similar resonance frequency (64 Hz) [20]. When tensile stress is induced in the spring (∆d = 0.1–0.5 mm), the resonance peak of the EVEH shifts towards higher frequency, because tensile stress increases the effective spring stiffness. In addition, the influence of the tensile stress on the output voltage amplitude of the EVEH is not obvious while tuning the resonance frequency. When compressive stress is induced in the spring, the resonance frequency first shifts towards lower frequency (∆d = −0.1 mm) and then shifts towards higher frequency (∆d = −0.2 mm and −0.3 mm). This phenomenon will be discussed with respect to Figure 10. The output amplitude first decreases (∆d = −0.1 mm and −0.2 mm) and then increases (∆d = −0.3 mm). Such a characteristic is induced by the combined effect of the increased spring stiffness from spring buckling and decreased coil-magnet distance.

Figure 10 shows the resonance frequency and coil-magnet distance as a function of the relative distance ∆d between the fixtures. The coil-magnet distance is constant (4.8 mm) when the tensile stress is introduced into the spring (∆d = 0.1–0.5 mm), because no buckling occurs with the presence of tensile stress. The resonance frequency increases from 88 Hz to 130 Hz when ∆d increases from 0.1 mm to 0.5 mm. The increase in the resonance frequency is a result of the enhanced effective spring stiffness for the bending mode of the spring. When the compressive stress is introduced into the spring, the coil-magnet distance decreases from 4.8 mm to 3.7 mm. Meanwhile, the resonance frequency first decreases from 88 Hz to 74 Hz and then increases to 115 Hz. The decrease in the resonance frequency is due to the reduced effective spring stiffness by the compressive stress and the subsequent increase in the resonance frequency is induced by the buckling. When the compressive stress increases beyond the buckling point, the excess length of the spring is relaxed by a central deflection, the stress will not increase anymore. Although compressive stress is known to decrease the spring stiffness, the buckling effect induced by the compressive stress is known to increase the effective spring stiffness, due to the dynamic axial force and the dynamic stretching of the springs. This behavior of successive decrease and increase in the resonance frequency induced by compressive stress is identical as the pattern predicted by Bouswtra and Geijselaers [21].

Table 2 lists the comparison between the present work and recent works of tunable energy harvesters based on both band broadening and frequency tuning techniques. For band broadening techniques, the multi-mode devices have rather narrow band width (often less than 10% of their resonance frequency) and the frequency band is the superposition of several narrow frequency bands [8,9]. The frequency tuning range of 64% in this work is larger than the bandwidth of the multi-mode devices. The multi-frequency device often utilizes array structures with different resonance frequency to expand frequency band. Despite the very large frequency band of ~1000 Hz [13]. The very high resonance frequency (~4000 Hz) deteriorates the applicability of such device in most of the ambient vibrations (1–200 Hz). For low frequency vibration energy harvesting, the non-linear [14], bistable [22], magnetostatic [23] wideband energy harvesters have the bandwidth between 5–19%, which is also lower than the relative tuning range of 64% in this work. These three types of devices have the potential to realize very large bandwidth, but each of them must overcome their inherent behavioral limitations before put into real applications, e.g., hysteresis for nonlinear devices, uncertain dynamic behavior under random and low vibrations for bistable devices and limitation of inter-magnet distance. For frequency tuning techniques, one of the most common techniques is to use magnetic force/coupling to tune the effective spring stiffness and, thus, the resonance frequency [20,24,25]. It is a simple and effective method, but its tuning range is limited by the distance between the magnets. Furthermore, the magnetic force increases rapidly when the distance between the magnets is small, giving rise to very limited output level at high frequency regime. The frequency tuning process can also be realized by adjusting the stress in the beam, which is often used in the piezoelectric energy harvesters [26,27,28]. Despite the effectiveness of tuning performance, the tuning structure used in these devices are often bulky, e.g., 1700 cm^3^ for a 6–62 Hz frequency range. Few works have been reported on the stress modulation in the spring of electromagnetic energy harvesters. In [29], the reported VEH device achieves a 65% frequency tuning range by applying an axial load on an aluminum spring with a central point mass. In this work, similar relative frequency tuning range is realized with a compact device with 28 times smaller volume compared to the VEH in [29]. It is worth mentioning that besides the common methods listed in Table 2, there are also other novel approaches to broaden the bandwidth of energy harvesters, such as using rotational pendulum [30], anti-resonance structure [31] and implementing the VEH in a non-resonating principle [32]. Compared with most of the works listed in Table 2, the tunable EVEH in this work has higher absolute and relative frequency tuning range, with a relative compact volume. However, before the proposed tunable EVEH can be used in real-world applications, the nonlinearity in the tuning performance must be addressed.

## 5. Conclusions

In this paper, we report an electromagnetic vibrational energy harvester with tunable resonance frequency. The resonance frequency of the EVEH is tuned by adjusting the axial stress introduced into the springs, which is realized by physically pulling and pushing the springs via a pair of metallic fixtures. The total tuning frequency range is 56 Hz (74–130 Hz), which is 64% of the natural resonance frequency of the EVEH (88 Hz). The tensile stress increases the resonance frequency of the EVEH, while the compressive stress firstly reduces the resonance frequency and then increases the resonance frequency due to buckling. In the future, new spring design might be implemented to improve the nonlinear relationship between the tuned resonance frequency and the tuning parameter (∆d). In addition, the tuning range of the resonance frequency might be further increased by optimizing the spring and fixture structure.

## Figures and Tables

**Table 1:** Design parameters of the EVEH.

Parameter	Description	Value
*E*	Young’s modulus	3.2 × 10^9^ Pa
*I*	Area moment of inertia, *I* = *bh*^3^/12	3.4 × 10^−16^ m^4^
*l*	Length of the spring	7.0 × 10^−3^ m
*b*	Width of the spring	1.0 × 10^−3^ m
*h*	Thickness of the spring	1.6 × 10^−4^ m
*ρ*	Weight per length	2.2 × 10^−4^ kg/m
*M*_T_	Weight of the tip mass	5.5 × 10^−3^ kg
*F*_cr_	Critical buckling load, *F*_cr_ = π^2^*EI*/l2	2.2 × 10^−1^ N

**Table 2:** Comparison between tunable energy harvesters.

	Reference	Technology	Size/cm^3^	Absolute Frequency Range/Hz	Relative Frequency Range/%
Band Broadening	[8]	Multi-mode device	1	71–74 105–107 111–114	4 2 3
	[9]	Multi-mode device	3.18	345–395 900–980 1130–1230	14 9 8
	[13]	Multi-frequency device, 40 cantilevers	1.40	3500–4500	25
	[13]	Multi-frequency device, 35 cantilevers	1.40	4200–5000	17
	[14]	Nonlinear device	9	94.1–98.9	5
	[22]	Bistable device	2.97	30–35	15
	[23]	Magnetostatic coupling device	68.9	45.5–55.5	19
Frequency Tuning	[24]	Combination of linear springs and magnetic nonlinearity	61	2–7	110
	[20]	Magnetic coupling adjustment of the spring stiffness	1.12	64–78	22
	[25]	Magnetic coupling adjustment of the spring stiffness	1.8	409–516	25
	[26]	Stress modulation of piezoelectric springs	1700	6–62	191
	[27]	Stress modulation of piezoelectric cantilever	54	292–380 440–460	22 4
	[28]	Stress modulation of a piezoelectric spring	60	200–250	24
	[29]	Stress modulation of an aluminum spring	280	16–46	65
	This work	Stress modulation of polymeric springs	9.95	74–130	64
